# Bridging the access gaps in HIV services for female sex workers who use drugs with person‐centred DSD models in Nairobi, Kenya: lessons learnt

**DOI:** 10.1002/jia2.26378

**Published:** 2024-10-18

**Authors:** Peninah Mwangi, Josephine Achieng, Beryl Abade, Janeffer Gacheru, Maureen Wanjiku, Daisy Kwala

**Affiliations:** ^1^ Bar Hostess Empowerment and Support Programme (BHESP) Nairobi Kenya

1

The Bar Hostess Empowerment & Support Programme (BHESP) was established in 1998 in Nairobi, Kenya, to provide a voice for women vulnerable to sexual and gender‐based violence to influence policy, reduce HIV acquisitions, support access to justice and reduce stigma and discrimination. BHESP operates for and by female sex workers (FSWs), women having sex with women and women using drugs and bar hostesses, many of whom live in informal settlements. BHESP engages their clients in HIV prevention, treatment and support services; gender and human rights awareness; legal services; advocacy and economic empowerment opportunities.

In 2020, BHESP observed that FSWs using drugs were alienated from accessing the current service delivery models due to community stigma, cultural and religious barriers. Consistent with BHESP's principles of community action, human rights and an evidence‐based response that puts the client at the centre of service delivery, FSWs who use drugs, peer educators, outreach workers, support group coordinators and clinicians were convened to lead the development, implementation and evaluation of tailored interventions to improve access for FSWs who use drugs. This was carried out in three parts: a community needs assessment; participatory processes and stakeholder consultations; and continuous monitoring and evaluation.

BHESP initiated this process by conducting a comprehensive community needs assessment with FSWs who use drugs to understand their diverse needs and challenges at each point of service delivery, including experiences of stigma, violence or geographic isolation (hidden sex workers). This individualized approach ensured that differentiated service delivery (DSD) models were tailored to the specific needs and circumstances of the FSW community.

BHESP organized community forums, focus group discussions and stakeholder meetings where FSWs and other key stakeholders, including clinicians, could contribute their perspectives, share experiences and co‐design solutions. By fostering collaboration and dialogue among diverse stakeholders, BHESP ensured that DSD models were informed by a holistic understanding of the social, cultural and structural factors influencing access to healthcare for FSWs who use drugs. The participants evaluated the unique individual needs of the clients and worked consultatively to come up with a mix of models that would best address those needs. This collaborative approach also enhanced the ownership and sustainability of DSD interventions within the community.

BHESP established robust monitoring and evaluation mechanisms to assess the effectiveness and impact of DSD models on the health outcomes and wellbeing of FSWs who use drugs. This involved tracking key indicators related to service utilization, health status and client satisfaction, as well as conducting regular assessments of programme implementation fidelity and quality. BHESP also solicited feedback from FSWs who use drugs and other stakeholders through surveys, focus groups and feedback forms to identify areas for improvement and adaptation. By continuously monitoring and evaluating DSD interventions, BHESP was able to identify emerging needs, gaps or challenges within the FSW who use the drug community and adjust approaches accordingly. This iterative process of learning and adaptation ensured that DSD models remained responsive to the evolving needs and preferences of FSWs who use drugs, ultimately enhancing the effectiveness and sustainability of the healthcare service delivery provided by BHESP.

Recognizing the intersectional nature of substance use within the FSW community, BHESP conducted targeted training sessions for healthcare providers, peer navigators and other stakeholders to raise awareness about the unique challenges faced by FSWs who use drugs and the importance of adopting a harm reduction approach. These sensitization efforts included workshops, seminars and peer‐led discussions that addressed stigma, discrimination and misconceptions surrounding drug use among FSWs. BHESP also facilitated dialogue between FSWs who use drugs and service providers to foster mutual understanding and empathy. Challenges in this process included entrenched stigma, resistance to harm reduction principles and misconceptions about drug use and sex work. However, through persistent advocacy and evidence‐based education, BHESP was able to gradually shift attitudes and perceptions among service providers, leading to increased acceptance and support for harm reduction interventions. The impact of the sensitization process was profound, as evidenced by improved access to non‐judgemental healthcare services, increased utilization of harm reduction tools such as needle and syringe programmes, opioid substitution therapy (methadone and buprenorphine), medication to prevent deaths from opioid overdose (naloxone) and safer sex supplies, and enhanced trust and collaboration between FSWs who use drugs and service providers. For FSWs who use drugs, opioid agonist therapy has been particularly beneficial as it provides a stable foundation for recovery, allowing them to focus on other aspects of their lives, such as addressing social and economic inequities contributing to their involvement in sex work. Ultimately, the sensitization efforts undertaken by BHESP not only addressed immediate barriers to care for FSWs who use drugs but also contributed to broader shifts in policy and practice within the healthcare system, paving the way for more inclusive and effective service delivery for other key and priority populations facing similar intersectional challenges.

This person‐centred and collaborative approach to the design of tailored interventions improved access to essential healthcare services for FSWs who use drugs and empowered them to actively participate in shared decision‐making processes affecting their health and wellbeing. The interventions developed via this participatory approach consisted of peer‐operated services, tailored clinic days and specific monthly theme days to advocate for gender‐responsive harm reduction services (see Table 1). It was recognized that a single service delivery model would not effectively address the diverse and individualized needs of all FSWs who use drugs. For example, there was a significantly higher uptake of opioid agonist therapy among clients who accessed services via the tailored clinic days. Service delivery was also adapted to virtual spaces during the COVID‐19 pandemic restrictions to ensure the continuation of services and has been maintained due to the ongoing need for virtual spaces. Since the implementation of these interventions in February 2021 until December 2023, 1214 newly identified FSWs who use drugs who had been until then disconnected from health services have accessed services among which 43 (3.5%) are living with HIV and 42 (98%) of these were successfully linked to antiretroviral therapy (ART), of which 37 (88%) are now virally suppressed (see Table 1 for results disaggregated by service type).

**Table 1:** Building blocks of BHESP person‐centred DSD models^1^ for female sex workers who use drugs and results from February 2021 to December 2023

	Peer‐operated services	Tailored clinic days	Theme days
*When is service provided*	Daily	Weekly	Monthly
*Where is service provided*	Community, virtual spaces	Drop‐in centre	Drop‐in centre, community, virtual spaces
*Who provides service*	Peer educator, social worker, field officer, adherence counsellor	Clients, healthcare workers, allies	Legal aid officers, civil society organizations, partner organizations
*What services are provided*	Legal representation, health welfare, social services, referrals and linkages, alcohol and drug services, sexual and reproductive health, including testing for HIV, hepatitis and sexually transmitted infections (STIs). Harm reduction, including opioid agonist therapy, needle and syringe distribution, mental health support and psychosocial assistance, tuberculosis screening and treatment	Facility‐based DSD which includes fast track antiretroviral (ARV) refills, HIV, hepatitis and STI testing, HIV prevention and psychosocial support Services can be tailored to the needs of specific groups, such as adolescent and young female sex workers or female sex workers who use drugs Harm reduction, including opioid agonist therapy, needle and syringe distribution, mental health support and psychosocial assistance, tuberculosis screening and treatment	Advocacy for gender responsive harm reduction services related to drug policy, dual stigmatization, human rights approaches to health and decriminalization Sexual and reproductive health services, including HIV, hepatitis and STI prevention, testing, treatment Harm reduction, including opioid agonist therapy, needle and syringe distribution, mental health support and psychosocial assistance, tuberculosis screening and treatment
*Number of newly identified clients accessing integrated services* ^a^	711	201	302
*Number of newly identified clients living with HIV (n[%])*	23 (3.2%)	7 (3.5%)	13 (4.3%)
*Number of newly identified clients living with HIV linked to antiretroviral therapy (ART) (n[%])*	22 (96%)	7 (100%)	13 (100%)
*Number of newly identified clients living with HIV linked to ART and who are virally suppressed (n[%])*	19 (86%)	7 (100%)	11 (85%)
*Number of newly identified clients prescribed or referred for pre exposure prophylaxis (PrEP) (n[%])*	114 (16%)	113 (56%)	164 (54%)
*Number of newly identified clients prescribed or referred for post exposure prophylaxis (PEP) (n[%])*	26 (3.7%)	4 (2.0%)	11 (3.6%)
*Number of condoms distributed to newly identified clients*	26,433	6432	9673
*Number of newly identified clients prescribed or referred for oral, implantable or injectable contraception (n[%])*	317 (45%)	177 (88%)	275 (91%)
*Number of newly identified clients tested for human papilloma virus (HPV) (n[%])*	0 (0%)	41 (20%)	16 (5.3%)
*Number of newly identified clients linked to cervical cancer screening (n[%])*	114 (16%) (Note: referrals only)	167 (83%) (Note: conducted at drop‐in centre)	218 (72%) (Note: conducted at drop‐in centre)
*Number of newly identified clients seeking support for alcohol use (n[%])*	589 (83%)	188 (94%)	271 (90%)
*Number of newly identified clients who accessed opioid agonist therapy (n[%])*	2 (0.3%) (Note: referral only)	27 (13%)	6 (2.0%)

^a^ Newly identified clients accessed at least one of the following integrated services including: testing for HIV, hepatitis and STIs; harm reduction services, including distribution of needles and syringes; TB screening; screening for gender‐based violence; mental health support services and socio‐legal support services.

During the planning and implementation of these interventions, BHESP conducted consultative feedback meetings, analysis of appointment management records and targeted gap analysis to identify barriers to effective service implementation. The challenges identified include:

1. Reluctance of service providers to access insecure locations such as injecting sites.
2. Transitioning of client‐provider relationships when healthcare workers leave BHESP.
3. Poor social determinants of health reflecting broader societal inequalities including unemployment, food scarcity, lack of transport, drug withdrawal or poor‐quality living environments.
4. Lack of context‐specific evidence, tools, standard operation procedures and client education materials to support the implementation.

Actions taken by BHESP to overcome these challenges included:

1. Using a one‐stop shop approach to the drop‐in centres and service delivery in the community, including the integration of psychosocial support services and harm reduction.
2. Encouraging clients to build relationships with multiple healthcare workers.
3. Providing legal aid clinics on theme days.
4. Providing ongoing education and training for providers on person‐centred care delivery, with a specific focus on holistic client needs assessment, management of symptoms and concerns, collaborative care planning and delivery.

To implement and achieve person‐centred DSD models within community settings, BHESP recommends:

1. Building relationships between clients and providers through effective communication and acknowledgement of clients as experts in their own healthcare that allow for sensitivity to client's values, needs and preferences.
2. Utilizing client‐reported outcomes to improve engagement and retention in care.
3. Supporting clients and providers to engage in shared decision‐making related to healthcare approaches.
4. Integrating person‐centred care in all service delivery points and additional services where needed, including tuberculosis prophylaxis, screening and treatment, sexual transmitted infection (STI) treatment, mental health interventions, overdose management, hepatitis B and C screening, alcohol and drug abuse screening and management, cervical cancer screening and referrals, pre exposure prophylaxis (PrEP), support services for people affected by violence, family planning and counselling aimed to assess and reduce vulnerabilities.

BHESP recommends community‐led, person‐centred DSD models specifically tailored for FSWs who use drugs as it individualizes client's management and improves their health outcomes. A mix of DSD models ensures that services are accessible and acceptable to FSWs who use drugs and address the unique challenges they face.

## COMPETING INTERESTS

The authors affirm that they have no competing interests relevant to the content of this work.

## AUTHORS’ CONTRIBUTIONS

This work is the result of collaborative efforts and extensive consultations among the authors, contributing to the comprehensive compilation of the manuscript.

## FUNDING

No external funding was received for the completion of this work or the preparation of this manuscript.

## Data Availability

Not applicable.

## Note

1 Updated recommendations on service delivery for the treatment and care of people living with HIV. Geneva: World Health Organization; 2021.

